# Tetraploidization of Immortalized Myoblasts Induced by Cell Fusion Drives Myogenic Sarcoma Development with *DMD* Deletion

**DOI:** 10.3390/cancers12051281

**Published:** 2020-05-19

**Authors:** Candice Merle, Noémie Thébault, Sophie LeGuellec, Jessica Baud, Gaëlle Pérot, Tom Lesluyes, Lucile Delespaul, Lydia Lartigue, Frédéric Chibon

**Affiliations:** 1 Institut National de la Santé et de la Recherche Médicale (INSERM) U1037, Cancer Research Center in Toulouse (CRCT), 31037 Toulouse, France; candice.merle@inserm.fr (C.M.); noemie.thebault@inserm.fr (N.T.); LeGuellec.Sophie@iuct-oncopole.fr (S.L.); gaelle.perot@inserm.fr (G.P.); tom.lesluyes@crick.ac.uk (T.L.); lucile.delespaul@icr.ac.uk (L.D.); 2 Department of Biology, University of Toulouse 3, Paul Sabatier, 118 route de Narbonne, 31062 Toulouse CEDEX 9, France; 3 Institut Claudius Régaud, IUCT-Oncopole, 31037 Toulouse, France; 4 Department of Pathology, Institut Claudius Régaud, IUCT-Oncopole, 31037 Toulouse, France; 5 Institut National de la Santé et de la Recherche Médicale (INSERM) U1218, 229 cours de l’Argonne, F-33076 Bordeaux, France; jessica.massiere@u-bordeaux.fr (J.B.); lydia.lartigue@gmail.com (L.L.); 6 Department of Life and Health Sciences, University of Bordeaux, 146 rue Léo Saignat, F-33000 Bordeaux, France; 7 Centre Hospitalier Universitaire (CHU) de Toulouse, IUCT-Oncopole, 31037 Toulouse, France; 8 Centre de Recherche en Cancérologie de Toulouse—Institut Universitaire de Cancérologie de Toulouse—Oncopôle (CRCT-IUCT-O), 2 Avenue Hubert Curien, 31037 Toulouse CEDEX 1, France

**Keywords:** cell fusion, genomic instability, sarcoma, dystrophin

## Abstract

Whole-genome doubling is the second most frequent genomic event, after *TP53* alterations, in advanced solid tumors and is associated with poor prognosis. Tetraploidization step will lead to aneuploidy and chromosomic rearrangements. The mechanism leading to tetraploid cells is important since endoreplication, abortive cytokinesis and cell fusion could have distinct consequences. Unlike processes based on duplication, cell fusion involves the merging of two different genomes, epigenomes and cellular states. Since it is involved in muscle differentiation, we hypothesized that it could play a role in the oncogenesis of myogenic cancers. Spontaneous hybrids, but not their non-fused immortalized myoblast counterparts they are generated from, induced tumors in mice. Unstable upon fusion, the hybrid genome evolved from initial mitosis to tumors with a highly rearranged genome. This genome remodeling finally produced targeted *DMD* deletions associated with replicative stress, isoform relocalization and metastatic spreading, exactly as observed in human myogenic sarcomas. In conclusion, these results draw a model of myogenic oncogenesis in which cell fusion and oncogene activation combine to produce pleomorphic aggressive sarcomas.

## 1. Introduction

A century ago, Theodor Boveri formulated the hypothesis that tetraploid cells are a transient stage during oncogenesis between the diploid and aneuploid states [1]. The most direct evidence for this was highlighted in early premalignant conditions such as Barrett’s esophagus, where a tetraploid state precedes aneuploidy, which coincides with the development of malignancy [2]. Furthermore, the population of 4N cells in ulcerative colitis corresponds to the early stage of the disease followed by aneuploidy [3]. Whole genome doubling is now known to occur in the early phases of many malignant phenotypes and is one of the most common events in cancer [4,5,6]. Boveri’s hypothesis has therefore received considerable support in pleomorphic sarcomas, i.e., a tetraploid stage likely occurs early in this disease [7,8,9]. One of the most important consequences of tetraploidization is the generation of aneuploid cells and chromosomal rearrangements [10,11,12].

Tetraploid cells may be generated in three ways: endoreplication, cytokinesis failure and cell fusion. Endoreplication is a mechanism in which cells increase their genomic DNA content without dividing [13] and contributes to the formation of megakaryocytes [14]. Cytokinesis failure occurs when there is a defect in the cytokinetic proteins or an error in chromosome segregation [15] Cell fusion generates heterokaryons that could lead to synkaryons, i.e., daughter cells with one nucleus. Cell fusion is a physiological process involved in fertilization [16], tissue regeneration [17] and myogenesis [18], and was already thought to contribute to tumor progression a century ago [19]. Since then, evidence has accumulated to prove this hypothesis. For many years, it has been shown to be involved in metastasis [20,21], in the generation of cancer stem cells [22] and to be a cause of tumor heterogeneity [23,24]. Whereas endoreplication and cytokinesis failure are the duplication of a single genome, cell fusion allow merging genome and properties of two different cells. We recently reported that spontaneous fusion between fibroblasts that are not fully transformed leads to tumorigenic hybrids displaying aneuploid patterns similar to those found in human pleomorphic sarcoma [25]. Furthermore, hybrid cells have been detected in animal models [26,27], in human tumors and in blood samples of patients, and are a predictive tool for disease stage and survival [26,27,28,29].

The fusion of myoblasts is the central mechanism for muscle differentiation, leading to a non-proliferating syncytium, thus ensuring the integrity of the genome. We hypothesized that this physiological mechanism could be diverted to proliferation upon activation of oncogenes. This tetraploidization state would lead to rearranged aneuploid genome that characterized pleomorphic sarcomas [30].

## 2. Results

### 2.1. Generation of Spontaneous Hybrid Cells and Phenotypic Characterization

**Figure 1:**
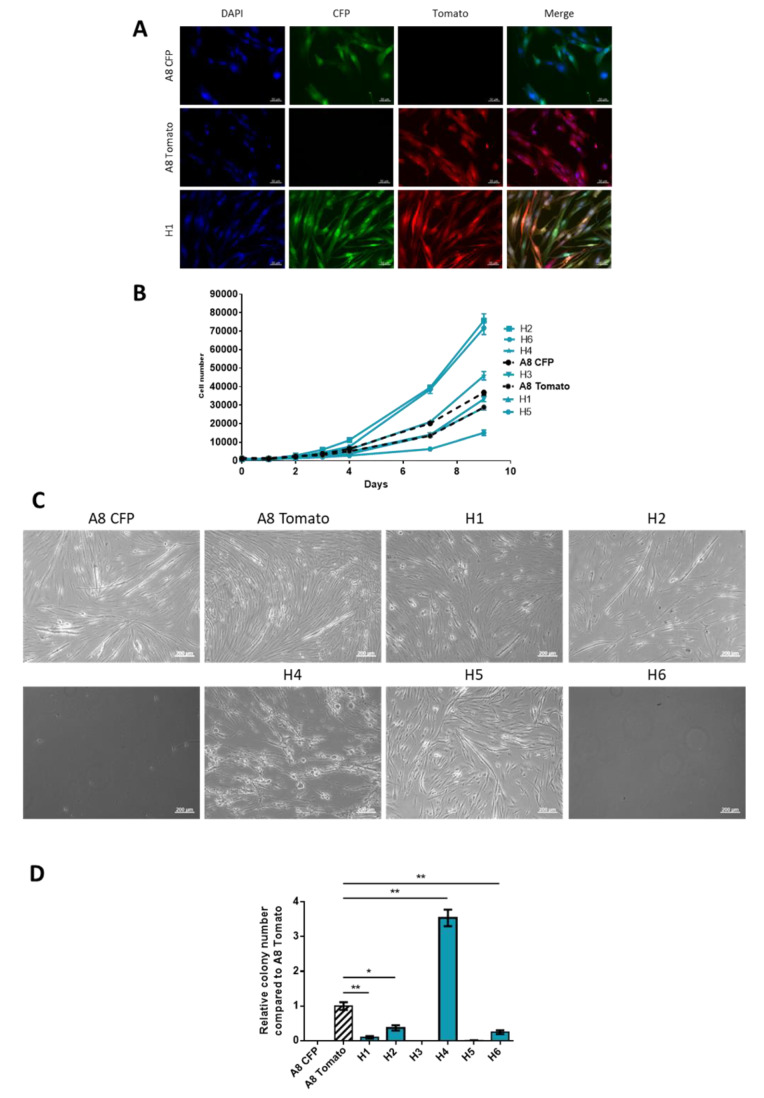
Validation of hybrids obtained by spontaneous cell fusion and phenotypic characterization. (**A**) Fluorescence expression of parental cell lines and H1. Scale bar = 50 µm. (**B**) Proliferation assay. Viable cell number was determined by flow cytometry from day 0 to day 9. Graph shows one representative experiment with triplicates for each cell line. This experiment was performed three times. (**C**) Evaluation of capacity to form myotubes. Images taken in phase contrast. Scale bar = 200 µm. (**D**) Capacity to form colony in soft agar (non-adherent conditions). Histogram shows one representative experiment with triplicates for each cell line. Experiment was performed three times. * *p*-value < 0.05; ** *p*-value < 0.01 Mann-Whitney test.

Two immortalized (by *CDK4* and *hTERT* introduction, see Material and Methods) human myoblast cell lines were transfected with plasmid CFP-Hygromycin (A8 CFP) or tdTomato-Blasticidin (A8 Tomato). After 72 h of co-culture, double antibiotic selection was added in order to select hybrids formed by spontaneous fusion. Six hybrid cell lines were established (H1 to H6) from distinct clones, each arising from one fusion event. All hybrids were mononucleated and expressed dual fluorescence, tdTomato and CFP, thus validating their hybrid nature (Figure 1A and Figure S1A,B).

To characterize the new properties acquired by the hybrids, we performed a series of phenotypic experiments. Parental and hybrid cell lines displayed the ability to proliferate heterogeneously. Half of the hybrids (H2, H6 and H4) showed an increase in proliferation capacity compared to the parental cell lines (Figure 1B).

Myoblasts are defined by their capacities to fuse to differentiate into myotubes under specific conditions. After 14 days of differentiation, parental cell lines and two hybrids, H1 and H5, had formed multinucleated myotubes (Figure 1C).

The other hybrids did not survive in these culture conditions (H2, H3 and H6) or formed round multinucleated cells (H4). A8 Tomato, H1, H2 H4 and H6 formed colonies in soft agar. However, apart from H4, hybrids did not form more colonies than the parental cell lines in non-adherent conditions (Figure 1D). In regards of these phenotypic properties, all hybrids cell lines display a heterogeneous behavior in vitro, most of the hybrids (H2, H3, H4, H6) harbor different characteristics compared to parental cell lines, such as loss of differentiation capacity or increase proliferation rate.

### 2.2. Hybrid Genomes Become Unstable upon Fusion and Evolves with Passages

**Figure 2:**
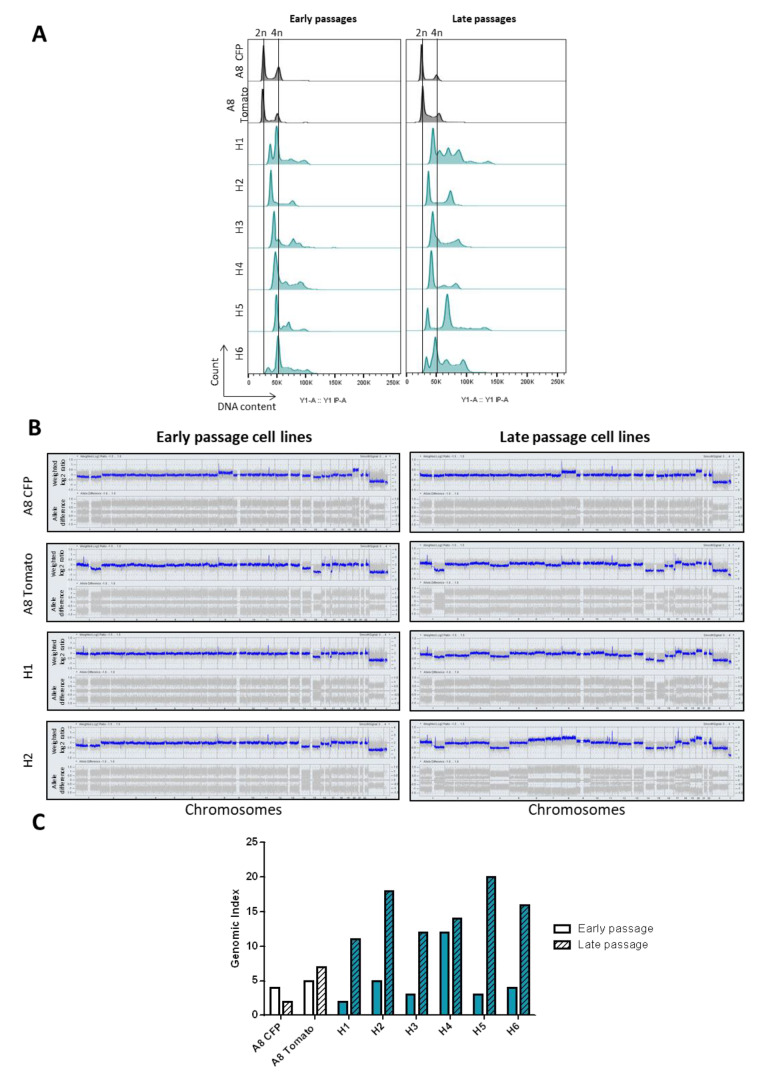
Hybrid cell lines present genomic rearrangements upon fusion and evolve with time. (**A**) FACS analysis of DNA content by measurement of fluorescent PI intensity. *x*-axis represents fluorescence intensity that is correlated with DNA content, *y*-axis represents the number of events detected. (**B**) Genomic profiling of parental and two hybrid cell lines at different passages. *x*-axis represents chromosome 1 to Y; *y*-axis represents CNV log2(ratio) (upper lane) and allele difference (lower lane). (**C**) Genomic index values of parental and hybrid cell lines at early and late passages. GI = A^2^/C where A corresponds to total number of alterations and C to number of chromosomes affected by these alterations.

To investigate the DNA content of hybrids, we first evaluated their ploidy (Figure 2A, left panel).

As expected, parental cell lines were diploids. Hybrid ploidy ranged from 3N (H2) to 4N (H6) and was heterogeneous in some clones (H1, H3, H4 and H6). To test whether the DNA content could evolve with passages, we also re-evaluated the ploidy at later passages (early evaluation: 13 to 20 passages vs late evaluation: 25 to 32 passages; Figure 2A, right panel). DNA content of parental cells was not modified with passages, but the ploidy of H1, H4 and H5 changed between early (EP) and late passages (LP). H1 lost its triploid population in favor of a tetraploid population and a small population of hexaploid cells emerged. On the other hand, the tetraploid population in H4 and H5 disappeared and triploid and hexaploid cells emerged, respectively. H2 and H3 were mostly triploid while H6 was tetraploid both at early and late passages.

Hybrid genomes were then analyzed by array CGH to characterize these ploidy modifications. The genomic profile of parental cell lines was not modified between early and late passages (Figure 2B). On the other hand, all hybrid genomes except H4 evolved with passages (Figure 2B and Figure S2A). They harbored extra copy number variations (CNVs) with losses and gains of the entire chromosome, which was correlated with a higher genomic index in the LP cell lines (Figure 2C). Penetrance plots highlight the main recurrent alterations found in EP and LP cell lines (Figure S2B). In EP hybrids, five chromosomes (chromosome 1q, 8, 14, 15, 17p, and X) were frequently altered (frequency higher than 50%). LP hybrids shared 12 alterations (more frequently losses than gains) and these alterations were different from those found in EP cell lines.

### 2.3. Tumor Development Occurs Only with Hybrids

**Figure 3:**
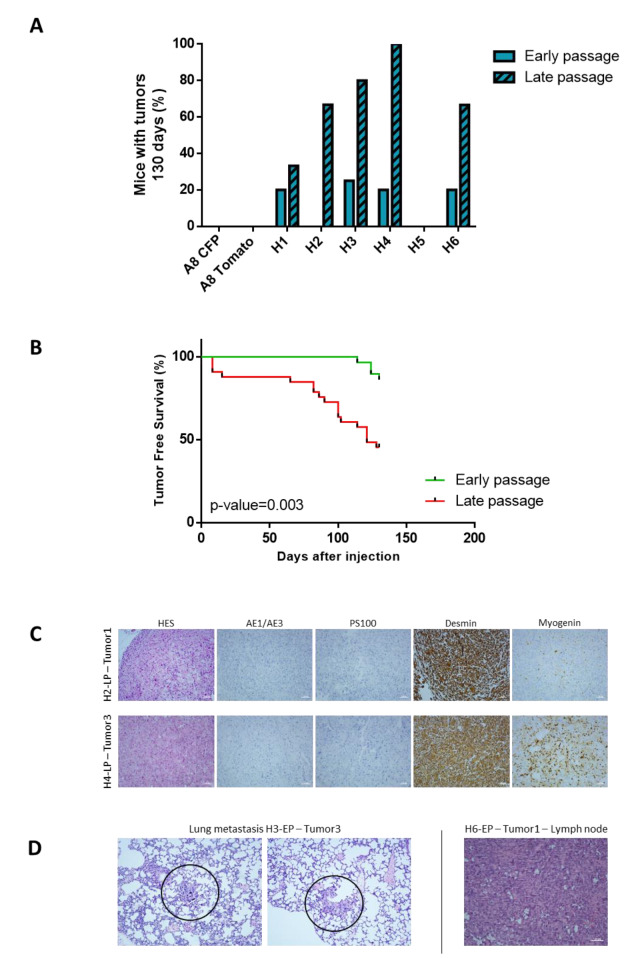
Hybrids develop tumors in mice and late passage cell lines are more aggressive. (**A**) Frequency of tumor development for parental and hybrid cell lines at early and late passage. (**B**) Tumor-free survival analysis comparing tumor development from early or late passage hybrid cell lines. *p*-value = 0.003 with Mantel-Cox test. (**C**) Hematoxylin and eosin (HE) staining and immunohistochemistry (IHC) analysis of hybrid tumors. Scale bar = 100 µm. (**D**) Lung and lymph node metastasis detected by HE staining. Scale bar = 100 µm.

Immortalized myoblasts are cell lines that do not develop tumors when injected in mice [31]. To test whether hybrids acquired tumorigenic properties after cell fusion, all parental and hybrid cell lines were injected in NSG mice both at early and late passages to test the impact of genome remodeling on tumor development. As expected, parental cell lines did not develop tumors after injection into mice, neither at early or late passage (Figure 3A and Figure S3A). Except H5, all hybrids developed tumors. All tumors were tdTomato- and CFP-positive, demonstrating that they arose from hybrids (Figure S3A). LP hybrids developed more tumors than those at early passages. At 130 days, 54% vs. 14% of mice injected with LP or EP hybrids, respectively, had developed a tumor, indicating that LP hybrids develop tumors more rapidly than EP hybrids (Figure 3B and Figure S3B).

Hybrid tumors were composed of pleomorphic cells with a large cytoplasmic compartment. Cells did not express Pan Cytokeratin AE1 and AE3 (carcinoma marker) and PS100 (melanoma marker) and had a strong expression of desmin and a focal expression of myogenin (Figure 3C and Figure S4B). All tumors were classified as pleomorphic rhabdomyosarcoma according to the WHO classification of soft tissue tumors [32]. Metastases were also detected in lungs for H3-EP and in lymph node for H6-EP (Figure 3D). In addition, tumoral emboli were identified for the H3-LP. The presence of these cells is usually a first step before metastatic dissemination [33] (Figure S4B). Cell lines from tumors were established wherever possible (see Material and Methods).

### 2.4. Hybrid Cell Lines Evolve towards a Genomic and Transcriptomic Pattern Promoting Oncogenesis

Results above show that LP cell lines developed tumors faster than EP ones and that hybrid cell lines evolve in culture conditions leading to cell lines with recurrent and specific chromosome number variations. Our hypothesis was that the genome of these LP cells is fit for oncogenesis, while EP cell lines need to acquire more instability to become competent for it. To test this, we studied the genome of tumors developed from EP and LP hybrid cell lines.

**Figure 4:**
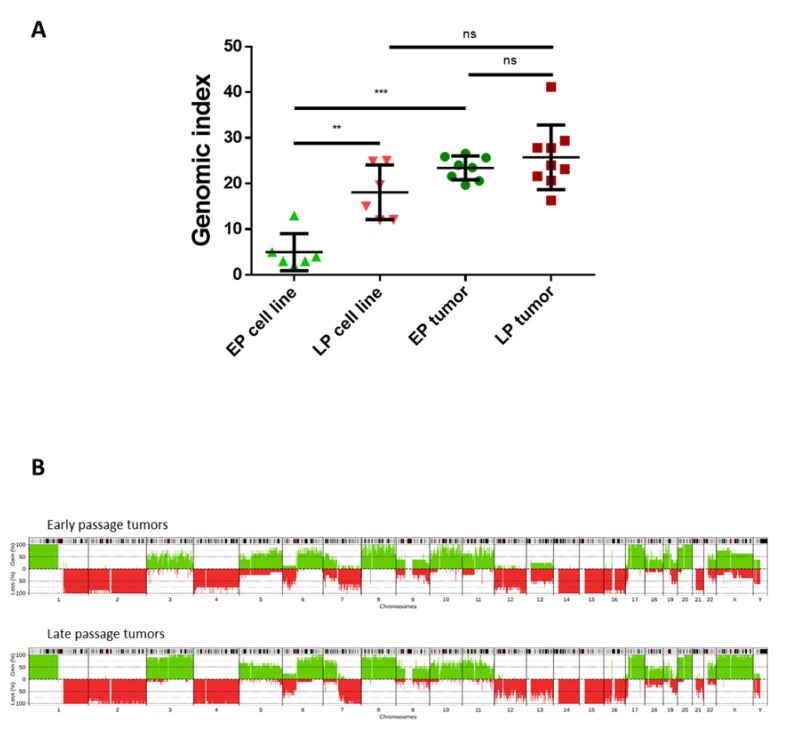
LP cell lines do not evolve in vivo whereas EP tumors display more chromosomic rearrangements than EP hybrids. (**A**) Comparison of genomic index between cell lines at EP and LP and their matching tumors. ns *p* > 0.05; ** *p* < 0.01; *** *p* < 0.001 Mann-Whitney test. (**B**) CNV frequencies (penetrance plot) in early (top) and late passage tumors (bottom). *x*-axis represents all chromosomes, *y*-axis represents proportion of tumors harboring chromosomal gains (green) and losses (red).

As expected, LP tumors did not have a different genomic index than the cell lines they derive from, so the genome did not harbor extra chromosomic rearrangements after in vivo engraftment (Figure 4A). In contrast, tumors developed from EP hybrids displayed more CNVs than cell lines in vitro, as reflected by an increase in the genomic index (Figure 4A and Figure S5). The genomic index of tumors developed by early and late passage cell lines was similar, indicating that they all reached the same level of rearrangement. Accordingly, penetrance plots showed that CNVs were very similar in EP and in LP tumors (gains of chromosomes 1p, 3, 5, 6q, 8, 10, 11, 17, 20, X; losses of chromosomes 1q, 2, 6p, 7q, 12, 14, 15, 16) with 54% of similarity (Figure 4B).

Since chromosomic alterations tended towards the same pattern after in vivo experiments with EP and LP cell lines, we hypothesized that all the cells moved to a specific genomic remodeling, resulting in a tumorigenic transcriptomic program. To test this hypothesis, we performed RNA sequencing of parental, EP and LP hybrid cell lines and three tumors from LP cell lines. PCA analysis separates samples into four groups: parental cell lines, EP cell lines, LP hybrids and cell lines derived from three tumors (Figure S6A). Then, we selected the genes that showed a correlation between their expression level and the stage of development, from parental cell lines to tumors (see Material and Methods). A total of 274 genes was found (Table S1). These genes clustered the samples into four groups (Figure S6B), mainly according to their status: parental (with both parental cell lines and H1 EP), EP hybrids (EP H2, H3, H5 and H6 with H3 LP), LP hybrids (H1, H2, H4, H5 and H6 LP with H4 EP) and tumors. Six groups of genes were identified. Groups 2 and 5 genes are highly expressed in parental cell lines and down-regulated in tumors and LP hybrids. Functional enrichment analysis with gene ontology showed that they are associated with the development process, cardiac morphogenesis and cell differentiation. Genes in group 3 had a opposite pattern with a low expression in parental and EP hybrids and a high expression in LP hybrids and tumors. They are associated with RNA processing/splicing. These results validate our hypothesis that upon cell fusion, the genome and transcriptome are remodeled toward oncogenesis.

### 2.5. Tumors Developed by Hybrids Present a DMD Deletion Specific to Myogenic Sarcomas

**Figure 5:**
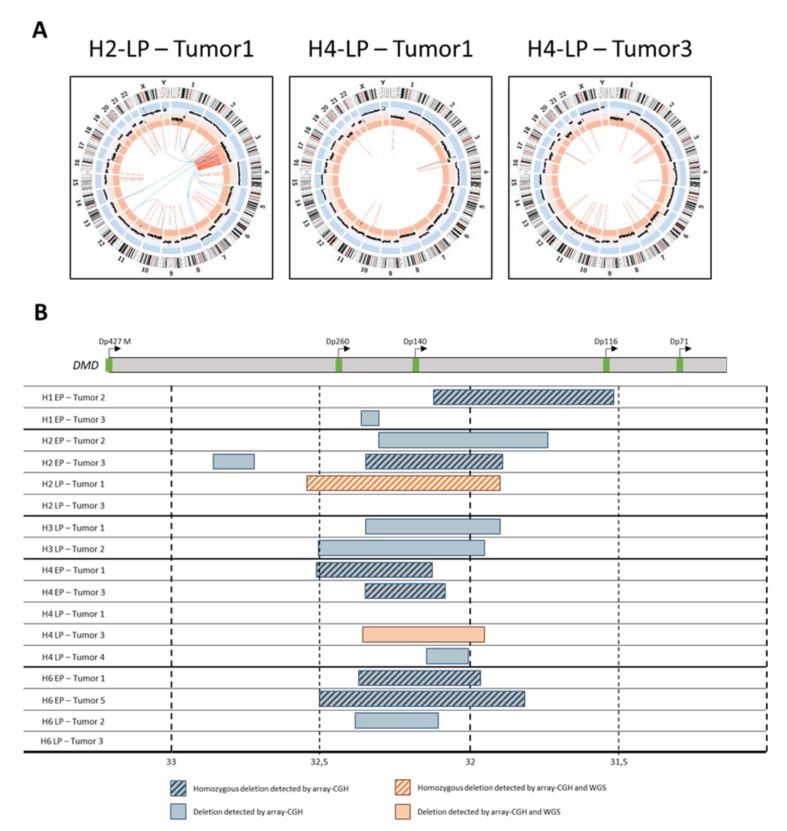
Hybrid tumors present complex chromosomic rearrangement including specific *DMD* deletions. (**A**) Circos plots representing chromosome ideogram, CNV and inter (blue) and intra-chromosomic (orange) structural variations. (**B**) *DMD* deletions overview of hybrid tumors. At top, a schematic representation of *DMD* transcripts. At bottom, blue boxes represent deletions detected by array CGH, and orange box deletions detected also by WGS and validated by PCR and Sanger sequencing.

Genome remodeling upon fusion was then analyzed by whole genome sequencing (WGS) in three samples. This highlighted three types of structural variants in addition to CNVs (Figure 5A): (i) intra-chromosomic translocations in all cases, (ii) inter-chromosomic rearrangements in two cases (H2-LP-Tumor1 and H4-LP-Tumor3) and (iii) chromothripsis along the entire chromosome 3 of H2-LP-Tumor1.

Array CGH analysis of hybrid tumors specifically evidenced focal recurrent intragenic deletions targeting *DMD* in 82% of cases (Figure 5B and Figure S7A), and occurring only after in vivo tumor growth. Half of the detected *DMD* deletions were homozygous. Since *DMD* inactivation by deletion has been reported to be a driver event in sarcomas with myogenic differentiation [34,35], we further characterized this highly frequent alteration. At the CGH level, deletion sizes and locations were different in all the tumors, including those within tumors developed from a same hybrid. Note that all deletions occurred in a region that affects Dp427, Dp260, Dp140 and Dp116 isoforms only, systematically preserving the 3′ end of the locus coding Dp71 isoform. Interestingly, this was reported in human sarcomas [34,35]. *DMD* deletion was detected in 2/3 samples subjected to WGS (H2-LP-Tumor1 and H2-LP-Tumor3), so we were able to define three fusion points in *DMD* and to validate these deletions by PCR and Sanger sequencing (Figure S7B). These deletions were found neither in parental cell lines nor in hybrids before engraftment, thus validating the CGH data (Figure S7B).

**Figure 6:**
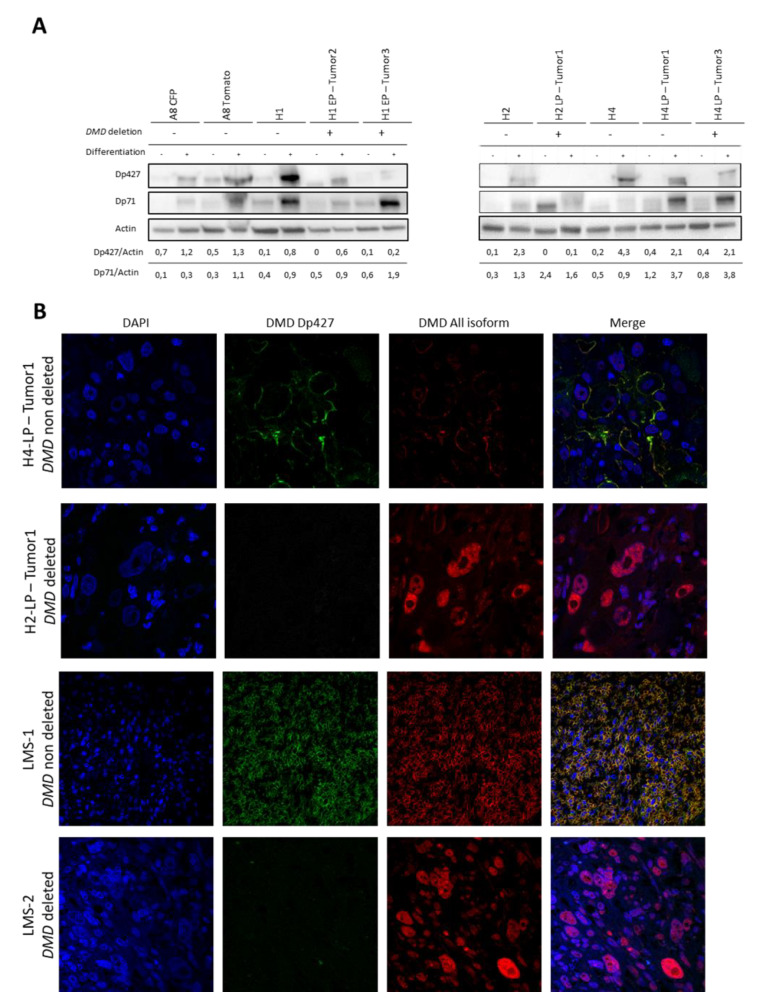
*DMD* deletions affect Dp427 isoform and lead to relocation of other isoforms. (**A**) Expression of Dp427 and Dp71 dystrophin isoforms by western blotting analysis in proliferation (−) or muscular differentiation (+) conditions. *DMD* deletion is indicated for each sample; −: *DMD* non deleted; +: *DMD* deleted. (**B**) Detection of Dp427 and other dystrophin isoforms by immunofluorescence analysis. Green fluorescence corresponds to Dp427 isoform, red to all dystrophin isoforms and blue is DAPI to detect nucleus.

Protein analysis (Figure 6A and Figure S8) showed that Dp71 expression was null or very low in proliferation conditions but increased in differentiation medium in all tumors, parental and hybrid cell lines tested, even in cases with *DMD* deletions. This result confirmed that the dystrophin isoform Dp71 is not targeted by these deletions. Samples were then classified into three groups depending on Dp427 isoform expression. First, all parental, all hybrids and hybrid tumors without *DMD* deletion (H4-LP-Tumor1) did not express Dp427, or only faintly, in proliferation conditions, whereas the expression increased in conditions of muscle differentiation. Second, tumors with the *DMD* deletion (H2-LP-Tumor1) did not express Dp427 in proliferation and differentiation conditions. The third group was composed of tumors that displayed a *DMD* deletion (H1-EP-Tumor2, H1-EP-Tumor3, H2-LP-Tumor1 and H4-LP-Tumor3) and maintained a weak expression of Dp427 in differentiation conditions. In this group, a heterozygous deletion (H4-LP-Tumor3) and/or a heterogeneous representation of *DMD* deletion in the tumor could be an explanation for the weak expression of Dp427.

*DMD* deletions did not target the Dp71 ORF and this isoform has been demonstrated to be essential for proliferation in myogenic cancers [34,35]. Our hypothesis was that the loss of the taller isoform will confer new properties to Dp71 protein by a modification of the localization of this isoform.

Accordingly, in the tumor H4-LP-Tumor1 (no *DMD* deletion), Dp427 (green) was detected by immunofluorescence on the FFPE tumor at the membrane of the cells as well as all the other isoforms (red) (Figure 6B). In samples that had a *DMD* deletion, H2-LP-Tumor1, H2-EP-Tumor2 and H6-EP-Tumor5, Dp427 was no longer expressed and all the other isoforms were strongly detected in the nucleus. H1-EP-Tumor2 and H3-LP-Tumor2 showed a heterogeneous and weak expression of Dp427, whereas all the other isoforms were mostly detected in the nucleus (Figure S9).

To sum up, when Dp427 was strongly expressed, it was located to the membrane with all DMD isoforms (H4-LP-Tumor1). In contrast, *DMD* deletions suppressed Dp427 isoform expression and all the other isoforms, including Dp71, were detected in the nucleus (H2-LP-Tumor1).

Because *DMD* deletion is thought to occur specifically in sarcomas with myogenic differentiation including leiomyosarcomas (LMS), we looked at the localization of the different isoforms of *DMD* in LMSs (Figure 6B and Figure S9). We selected tumors with and without *DMD* deletion in a previously characterized and reported cohort of LMSs [35]. As expected, LMSs without *DMD* deletion strongly expressed Dp427 and all the other isoforms were exclusively in the cell membrane. On the other hand, when *DMD* was deleted, Dp427 was no longer expressed and all the other isoforms were relocated in the nucleus.

## 3. Discussion

By testing whether cell-cell fusion is involved in the oncogenesis of myogenic sarcoma, we found that cell fusion generates hybrids promoting tumor initiation and metastasis. After cell fusion, the genome of proliferating hybrids evolves until the cells are fit for oncogenesis. In vivo, upon selection pressure, emerging tumors very frequently harbor *DMD* deletions similar to those described in human myogenic sarcomas. These deletions abrogate Dp427 isoform expression and trigger the relocation of the other isoforms, including Dp71, to the nucleus.

Myoblasts with the oncogenes *CDK4* and *hTERT* do not develop tumors in mice [31], which means that the combination of these two oncogenes is not sufficient for tumor inception. In our previous study [25], we demonstrated that cell fusion merges activated oncogenes from two different cells to produce a hybrid cell with the right oncogenic combination needed for tumor development. More importantly, cell fusion also induced genomic instability. In the myoblast model reported here, cell fusion did not combine complementary oncogenes (both parental cells harbored the same oncogenes) but led through mitosis to genome merging and a transient tetraploid state followed by aneuploidy, and ultimately to genomic instability. The involvement of cell fusion in oncogenesis expands the hypothesis that aneuploid cancer cells might develop from a tetraploid intermediate. The first consequence of cell fusion, like endoreplication and cytokinesis failure, is the generation of tetraploid hybrid cells which harbor extra-centrosomes, leading to multipolar mitosis and genomic instability [36]. Compared to other mechanisms that lead to 4N cells, cell fusion is also the merging of two cells with different cell cycles, epigenomes and differentiation stages. This confrontation of two distinct statuses could increase the chromosomic instability generated by the tetraploid state and further lead to cells with a more rearranged genome. *CDK4* overexpression alters the pRb pathway, allowing the generation, proliferation and viability of tetraploid cells. One hypothesis of oncogenesis is that the pRb pathway deregulation is the first event, followed by cell fusion that induces a transient tetraploid state followed by aneuploidy and chromosomic instability. Finally, the genome is remodeled for tumor development.

We posit three phases of genome remodeling after cell fusion. First, the alterations found in each parental cell line are merged in the hybrid cells. Second, the hybrids acquire large new copy number variations, with losses and gains of arms or entire chromosomes. These variations are not random because they are shared by a high proportion of the hybrids and are generated in two different environments, in vitro (for the late passage hybrids) and in vivo (for the early passage hybrids). It is likely that these specific losses and gains are necessary for tumor development in this specific myogenic context [37]. All the hybrid cells evolved similarly, leading to cells competent for tumor inception and recapitulating Darwin’s theory that events are gradually acquired and that cells which are the most suited to a specific interaction cell/environment are selected. Once the cells become suitable, they do not acquire extra copy number variations, as observed in the engraftment of late passage hybrids.

The third phase of genome evolution might correspond to the acquisition of punctual focus alterations, i.e., 5′-*DMD* deletions, likely related to expression/replication conflict under in vivo pressure selection. This kind of alteration has been described as “replicative stress” [38,39]. The progressive accumulation of these different types of alterations, CNVs and SVs (structural variations), are evocative of what was recently found in human tumors. In the TRACERx project, the authors described aggressive tumors that accumulate oncogenic drivers in primary tumors, leading to metastasis that is more homogeneous than their tumor counterparts [6,40]. Progressive acquisition of these alterations is correlated with an evolution of the transcriptomic program, from parental cells to tumors. Thus, the expression of genes in development and cell differentiation decreased from parental myoblasts to hybrid tumors. This is reminiscent of human rhabdomyosarcomas where markers of late differentiation are not expressed and the cells are not able to differentiate and gain the capacity to proliferate leaving their quiescent state [41]. Genes associated with RNA splicing are overexpressed in hybrid tumors compared to parental cells. Splicing factor upregulation and aberrant splicing are both common in cancer [42,43] and could account for the expression of specific isoforms related to differentiation and proliferation.

The development of myoblast hybrid tumors is associated with highly frequent *DMD* structural alteration (deletions). *DMD* is one of the longest human genes (2.2 Mb). Spatial and temporal overlap of transcription and replication machineries in the late S phase triggers DNA break spots called common fragile sites (CFSs), particularly in long genes [38]. This instability is related to gene expression. Dp427 is not expressed in normal myoblasts and its expression is found only in differentiated fibers that do not proliferate [44]. We show here that in conditions of differentiation induction, cycling hybrid cells do not form myotubes but express the Dp427 isoform. Thus, the imbalance conflict between cell cycle (replication) and differentiation (transcription) promotes the crash between replication and transcription machineries, ending up with deletion in highly expressed and late replicating genes. *DMD* deletions are then detected because the locus fits these features and deletions have a tumor suppressor impact on oncogenesis [35].

Deletion of *DMD* abrogates the expression of the Dp427 isoform, always preserving Dp71. Even if we cannot definitively rule out the possibility that cell cycle status could account for Dp71 delocalization this is unlikely since we observed, both in hybrid cell lines and in human tumors, that Dp427 loss is strongly associated to Dp71 nucleus relocation. Dp427 is a tumor suppressor [34] whereas the inhibition of Dp71 leads to a decrease in proliferation and to cell cycle arrest [35], demonstrating that this isoform is essential to tumor cells. Deletion of Dp427 seems to confer new properties to Dp71 in a sarcoma context, hypothesizing that Dp71 isoform could have a role in metastasis development [34,35]. In early myogenesis, Dp71 is expressed and then downregulated in mature muscle fibers, allowing Dp427 membrane expression and interactions with Dystrophin Associated Protein Complex (DAPC) [45]. Dp71 isoform have already been described in the nucleus of HeLa cells [46] and is linked to β-dystroglycan and α-sarcoglycan which are anchored in the nuclear membrane [47].One of the hypotheses on the oncogenic role of Dp71 triggers by Dp427 deletion is that Dp71 and DAPC could have the same function in the nucleus membrane as Dp427 and DAPC in the cellular membrane, thereby participating in the regulation of nuclear processes by modifying the nucleus membrane. Because *DMD* deletion is associated with metastatic evolution [35], data suggest that by triggering the relocation of Dp71 to the nucleus, it provides it with new properties in favor of oncogenesis.

In conclusion, cell fusion, the event involved in myoblast differentiation is a way to induce in a single event genomic remodeling and genetic aberrations leading to tumor development. Together with oncogene activation upon mutation, myoblast fusion generates a model reflecting the evolution of the genome following whole genome doubling and could account not only for the aneuploidy commonly found in pleomorphic sarcoma but also, and more specifically, for the targeted genomic alterations specific to histotypes.

## 4. Material and Methods

### 4.1. Cell Lines and Culture Conditions

Immortalized human myoblast cell lines (*CDK4* and *hTERT*) were kindly provided by Bénédicte Chazaud. Immortalization of human myoblast were performed as previous described [31]. Cell lines were cultured in DMEM supplemented with 20% heat-inactivated fetal bovine serum (FBS), 10% medium 199 and 30 ng/mL of dexamethasone (Sigma-Aldrich, St. Louis, MO, USA) at 37 °C with 5% CO_2_. Parental cell lines were generated by lentiviral infection using hygromycin-CFP and blasticidin-tdTomato plasmids (kindly provided by Dr. Richard Iggo). Tumor cell lines obtained from xenografts were obtained after 24 h of collagenase treatment and then cultured in the medium described above. Passages of cell lines described as “early passages hybrid” range from passages 13 to 20 after selection of clones; passages of cell lines described as “late passage hybrids” range from passages 25 to 32.

### 4.2. Generation and Validation of Hybrid Cell Lines

Parental cell lines were seeded in a six-well plate at a ratio of 1:1 with 150,000 cells per well. After 72 h, spontaneous hybrids were selected by adding blasticidin (18 µg/mL, ThermoFisher Scientific, Waltham, MA, USA) and hygromycin (12 µg/mL, Sigma-Aldrich). To validate hybrid selection, dual fluorescence was evaluated by microscopy (Cell Observer Microscope, Zeiss, Oberkochen, Germany) and flow cytometry (MACS Quant VYB, Miltenyi, Bergisch Gladbach, Germany).

### 4.3. Ploidy Evaluation by Flow Cytometry

Cell lines were resuspended in PBS 1X and fixed with 70% ethanol overnight at −20 °C. Then, cells were washed and a solution of propidium iodide (Sigma Aldrich, 50 µg/mL) and RNAse A (ThermoFisher Scientific, 10 µg/mL) was added for 30 min at room temperature. DNA content was quantified with propidium iodide fluorescence intensity by flow cytometry (MACS Quant VYB, Miltenyi) and analyzed using FlowJo software (BD Bioscience, Franklin Lakes, NJ, USA).

### 4.4. Proliferation Assay

In triplicate, 1000 cells were seeded in a 96-well plate. From day 1 to day 9, cells were harvested and collected in 200 µL of FACS buffer (PBS 1× supplemented with 5% FBS and 2mM EDTA). The number of viable cells was determined by flow cytometry (MACSQuant^®^ VYB, Miltenyi, Bergisch Gladbach, Germany) and data were analyzed using FlowJo (BD Bioscience).

### 4.5. Muscular Differentiation Assay

Cells were seeded at 500,000 cells in a six-well plate. Cells were cultured for 14 days in differentiation medium with DMEM, insulin (Sigma Aldrich, I2643, 1 mg/mL) and transferrin (Sigma Aldrich, T8158, 50 mg/mL). Medium was changed every two days during the first week and then every day. At 14 days, cells were fixed with 4% PFA and images were taken with Axio Vert.A1 (Zeiss). For western blot and immunofluorescence in muscular differentiation conditions, cells were cultivated for 6 days in differentiation medium.

### 4.6. Soft agar Colony Formation Assay

Cells were diluted in 0.35% agar medium solution in order to seed 5000 cells per well. A mix of agarose and cells was left on the top of pre-coated 0.5% agarose. Medium was changed once a week. After 6 weeks, colony number for one field was manually counted using an Axio Vert.A1 (Zeiss, Oberkochen, Germany) at 4x magnification. Each cell line was seeded in triplicate and two fields were counted for each well.

### 4.7. In Vivo Experimentations

Animals were maintained under specific pathogen-free conditions in the animal facility of Bordeaux University, and under opportunist and pathogen-free conditions in the CREFRE US006, Toulouse. Experiments were performed in conformity with the rules of the Institutional Animal Care and Use committee (approval number 362 DIR13109 and DAP-APAFiS-201802161802878) and all efforts were made to minimize animal suffering.

One million cells in 100 µL PBS 1× were subcutaneously injected into 8-week-old NSG mice. For early passage cell lines, five mice were injected and for late passage cell lines six mice were injected. Tumors were measured every week using a digital caliper and tumor volume was calculated using the formula V = (length × width^2^)/2. Once tumors reached 2000 mm^3^, mice were sacrificed by cervical dislocation. Tumors were formalin-fixed and nitrogen-frozen, and one part was also used to establish cell lines from the tumor. Survival curves were plotted with Graph Pad Prism (Graph Pad Software, San Diego, CA, USA)) and statistical analysis was performed with the Log-rank Mantel-Cox test.

### 4.8. HE Staining and Immunohistochemistry

A 4 µm-thick section of formalin-fixed paraffin-embedded tumor bloc was used for HE staining and immunochemistry. HE staining was performed according to standard protocols. Immunohistochemistry (IHC) was performed using the mouse monoclonal antibody against human protein S100 (clone Z311, Agilent Technologies, Santa Clara, CA, USA), myogenin (clone LO26, Novocastra, Leica Biosystems, Wetzlar, Germany), desmin (Dako clone D33, Agilent) and pan-cytokeratin (clone AE1/AE3, Agilent). Pictures were captured using a Zeiss Cell Observer Microscope.

### 4.9. Tissue Immunofluorescence

A 4-µm formalin-fixed paraffin-embedded tissue section was de-paraffinized in three xylene baths for 5 min and then rehydrated in successive baths of ethanol from 100% to 70%. For heat-induced epitope retrieval, slides were incubated for 20 min in a microwave oven in Dako Target Retrieval pH6. Then, they were incubated with primary antibodies for 1 h at room temperature in a humidity chamber and after 1 h with secondary antibodies. Slides were then mounted with Vectashield mounting medium plus DAPI (Vector Laboratories, Burlingame, CA, USA). Images were acquired on a Zeiss Cell Observer Microscope or a LSM 780 confocal microscope (Zeiss). Primary antibodies used for Dp427 were NCL-DYS1 (1/25, Novacastra, Leica, Wetzlar, Germany) and ab15277 for all the dystrophin isoforms (1/100, Abcam, Cambridge, UK). Secondary antibodies used were Alexa Fluor 488 (ThermoFisher Scientific, Waltham, MA, USA, 1/400) anti-mouse and Alexa Fluor 594 anti-rabbit (ThermoFisher, 1/400).

### 4.10. RNA Extraction, Sequencing and Analysis

Total RNA from cell lines was extracted with TRIzol reagent (Life Technologies) and purified with the RNeasy MinElute Cleanup Kit (Qiagen, Hilden, Germany) according to the manufacturer’s instructions. RNA concentration was evaluated with Clariostar and quality-checked with an Agilent 2100 Bioanalyzer (Agilent Technologies). RNA sequencing was performed by the Centro Nacional de Análisis Genómico (CNAG, Barcelona, Spain) to obtain more than 20 million paired-end reads with a length of 75 bp. Subsequent CNAG RNA sequencing steps, ERCC spike-in sequencing control quality check and bioinformatics analysis pipeline have already been described [48]. Thereafter, genes with a FPKM mean across all cell lines superior to 1 were used to conduct a principal component analysis (PCA) using R package FactoMineR. Gene expression was evaluated for the following groups: (1) A8 CFP and A8 Tomato, (2) H2 EP, H4 EP and H6 EP, (3) H2 LP, H4 LP and H6 LP, 4) H2 LP Tumor 1, H4 LP Tumor 3 and H6 LP Tumor 2. Then, Pearson’s correlation was established from group 1 to group 4. Genes with a Pearson’s coefficient ranges between 0.9 to 1 and −0.9 to −1 were selected to make a cluster analysis of z-score gene expression (https://github.com/obigriffith/biostar-tutorials/blob/master/Heatmaps/heatmap.3.R). Finally, six groups of genes were identified and a functional enrichment analysis of gene ontology was performed using Bioconductor packages goseq and org.Hs.eg.db.

### 4.11. Genomic DNA Extraction, Cytoscan HD Array and Genomic Index Calculation

DNA extractions were performed on cell lines and frozen tumor samples. Genomic DNA was extracted using the standard phenol-chloroform method and quantified using Clariostar. DNA arrays were performed using Affymetrix CytoScan HD Arrays (Affymetrix, Santa Clara, CA, USA) according to the manufacturer’s instructions. Analysis was performed using the Chromosome Analysis Suite software (Affymetrix) with genome version GRCH37 (hg19). Genomic index was calculated as follows: GI = A²/C where A corresponds to the total number of alterations and C to the number of chromosomes affected by these alterations. To compare genomic index of the different groups, a Mann-Whitney test was performed. Losses and gains of each sample were defined with an average log2(ratio) (Smooth signal) ≤ −0.2 or ≥ 0.2, respectively. Thus, penetrance plots were obtained for four subgroups: EP hybrids, LP hybrids, EP tumors and LP tumors.

### 4.12. Whole Genome Sequencing and Structural Variant Analysis

DNA of H2 LP Tumor 1, H4 LP Tumor 1 and H4 LP Tumor 3 were sequenced at CNAG. Whole genome sequencing characteristics were paired-end sequencing with 150 bp reads and an expected coverage of 45×. Sequenced reads were aligned on Human GRCh38/hg38 genome with BWA mem v0.7.17 [49] and PCR duplicates were removed with MarkDuplicates (PicardTools v2.18.2, http://broadinstitute.github.io/picard/). Thus, genomic rearrangements were established with GRIDSS [50] v2.0.0 and filtered to keep those occurring in one tumor sample, using bcftools (https://samtools.github.io/bcftools/bcftools.html) v1.6 with RAS > 0, AS > 0 and QUAL > 0 criteria.

A Circos plot [51] was performed with array-CGH average log2ratio and structural variants.

### 4.13. PCR

For PCR, primers were designed using the Primer 3 program (http://frodo.wi.mit.edu.proxy.insermbiblio.inist.fr/primer3/). The touch-down (TD) 60 °C program was used (TD 60 °C; 2 cycles at a 60 °C, followed by 2 cycles at 59 °C, 2 cycles at 58 °C, 3 cycles at 57 °C, 3 cycles at 56 °C, 4 cycles at 55 °C, 4 cycles at 54 °C, 5 cycles at 53 °C, and finally 10 cycles at 52 °C). PCR was performed on 50 ng of DNA using AmpliTaqGold^®^ DNA polymerase (ThermoFisher Scientific).

### 4.14. Western Blotting

Cells were rinsed in PBS 1× and lysed for 20 min at 4 °C in RIPA buffer (R0278, Sigma Aldrich) supplemented by phosphatase/protease inhibitor cocktail (1×, Sigma Aldrich). Proteins were collected in supernatant after 15 min of centrifugation at 13,000g and quantified by DC protein assay kit, Biorad. 40 µg of total proteins were loaded on Mini Proteane TGX stain free 4–15% (BioRad, Hercules, CA, USA). Transfers on PVDF membrane were performed using a dry transfer system (iBlot2, ThermoFisher Scientific) and membranes were blocked in 5% non-fat dry milk in 0.1% PBS-Tween solution. Membranes were incubated overnight with the primary antibody: mouse anti-Dp427 (NCL-DYS1, Leica, 1/200) or Rabbit anti-Dp71 (Ab15277, Abcam, 1/500) at 4 °C overnight. After wash, membranes were incubated for 1 h at room temperature with appropriate secondary antibodies: anti-rabbit HRP (7074S, 1/1000, Ozyme, Saint-Cyr-l’Ecole, France) and anti-mouse HRP (7076S, 1/1000, Ozyme). After incubation with chemiluminescent substrate (ECL Immobilon Western, WBKLS0100, Merck, Darmstadt, Germany), signals were detected using PXi (Syngene, Cambridge, UK).

## 5. Conclusions

In conclusion, cell fusion is a way to induce in a single event genomic remodeling and genetic aberrations leading to tumor development and metastasis progression. This tetraploidization mechanism, with oncogene activation, permits to reflect the evolution of myogenic sarcoma, from aneuploidy to genomic alterations specific to this histotype.

## Supplementary Materials

The following are available online at https://www.mdpi.com/2072-6694/12/5/1281/s1, Figure S1: Fluorescence analysis, Figure S2: Genomic analysis of hybrid cells lines at early and late passages shows evolution of genomes, Figure S3: Late passage cell lines developed more tumors than early passage cell lines, Figure S4: Characterization of hybrid tumors developed in mice, Figure S5: Genomic profiling, Figure S6: Transcriptomic program is remodeled toward oncogenesis, Figure S7: Identification and validation of *DMD* deletions in hybrid tumors, Figure S8: Signal detection on the whole membrane shown on Figure 6A, Figure S9: Detection of Dp427 and other dystrophin isoforms by immunofluorescence analysis, Table S1: Genes used to cluster the samples into four groups.
