# Supplementary material for: Tetraploidization of Immortalized Myoblasts Induced by Cell Fusion Drives Myogenic Sarcoma Development with DMD Deletion
Source: Cancers (Basel). 2020 May 19;12(5):1281. doi: 10.3390/cancers12051281 (PMC7281535; doi:10.3390/cancers12051281)
Supplement: Supplementary file 1 [file cancers-12-01281-s001.pdf]

Supplementary Materials: Tetraploidization of Immortalized Myoblasts Induced by Cell Fusion Drives Myogenic Sarcoma Development with *DMD* Deletion

Candice Merle, Noémie Thébault, Sophie LeGuellec, Jessica Baud, Gaëlle Pérot, Tom Lesluyes, Lucile Delespaul, Lydia Lartigue and Frédéric Chibon

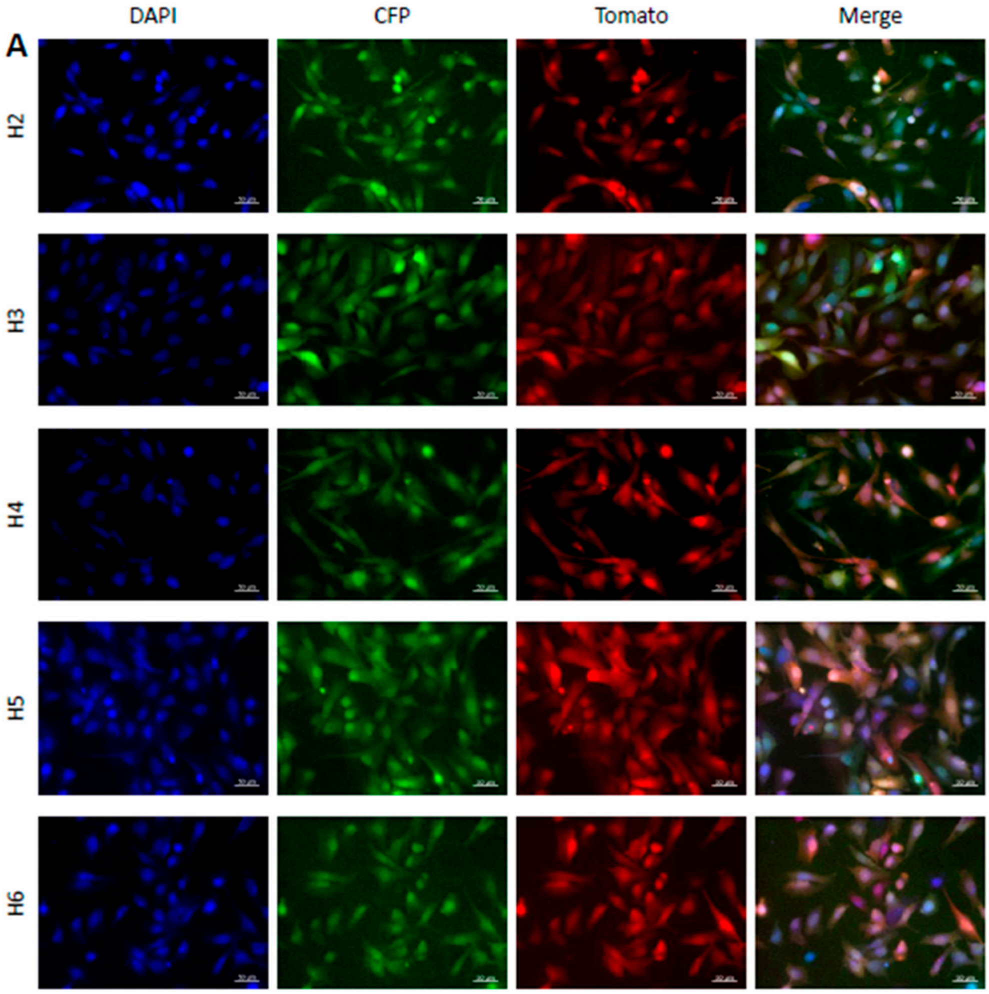

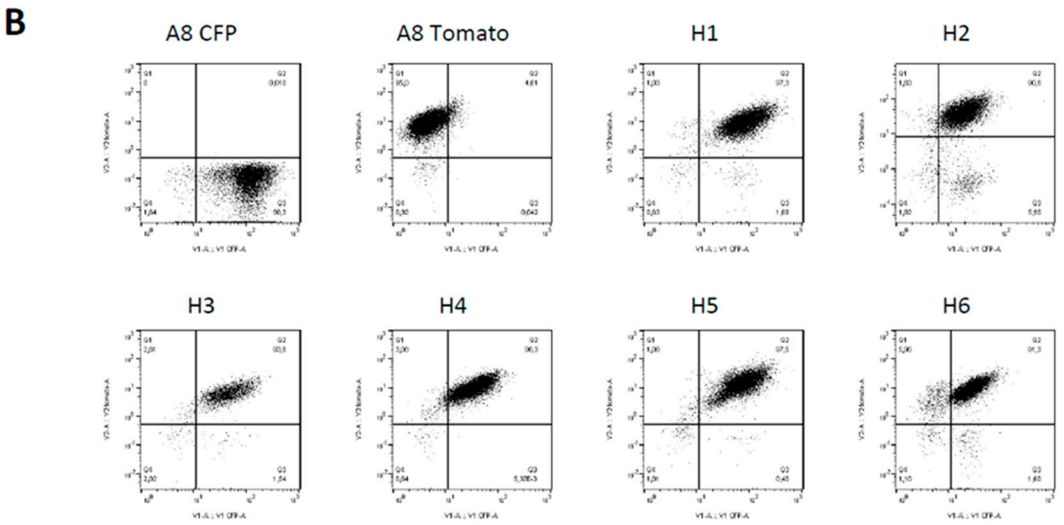

Figure S1. Fluorescence analysis. (**A**) Fluorescence expression of hybrid cell lines H2 to H6. Scale bare = 50 µm. (**B**) Flow cytometry analysis of CFP (x-axis) and tdTomato (y-axis) fluorescence expression in parental and hybrid cell lines.

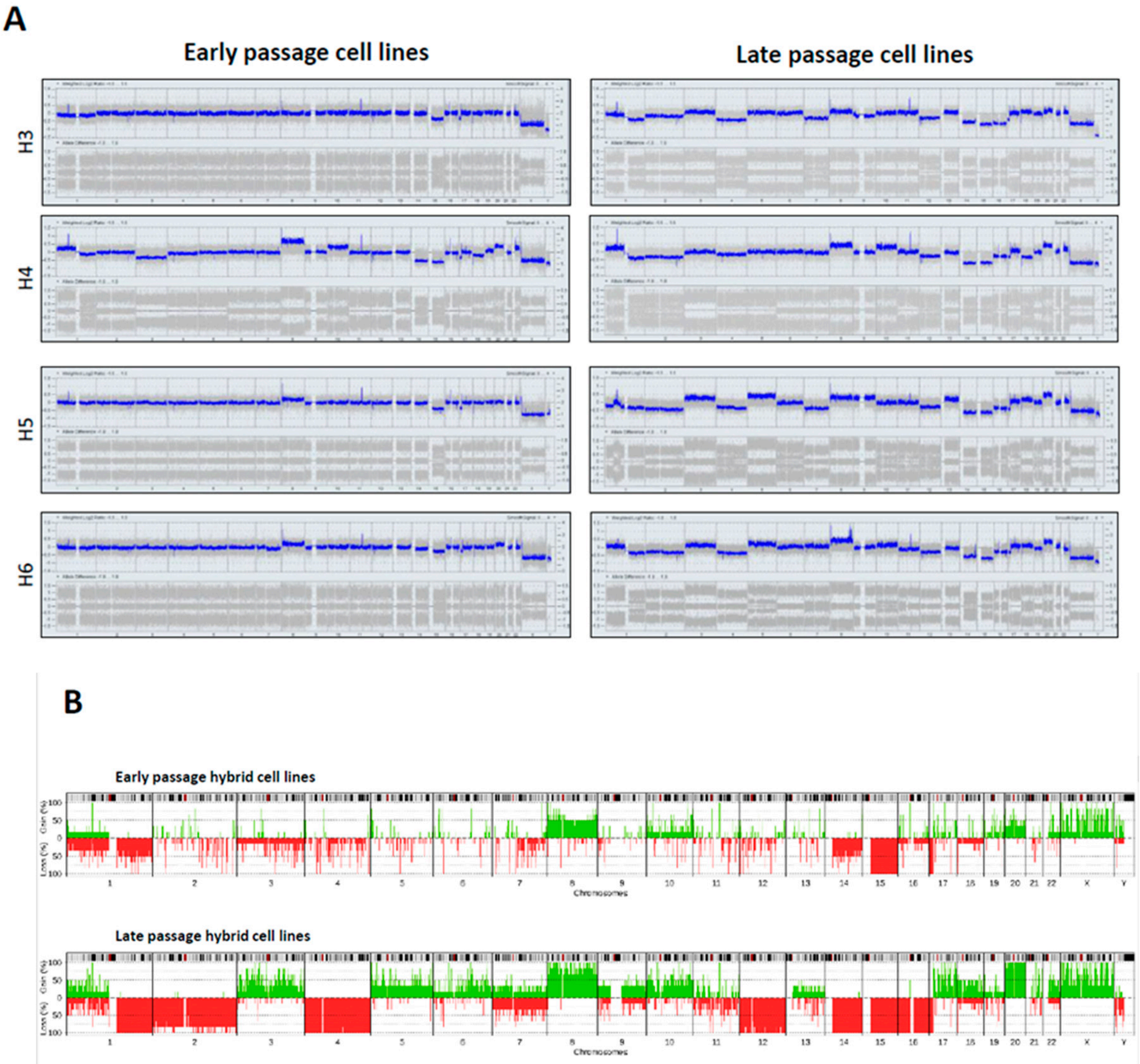

Figure S2. Genomic analysis of hybrid cells lines at early and late passages shows evolution of genomes. (**A**) Genomic profiling of parental and two hybrid cell lines at different passages. x-axis represents chromosome 1 to Y; y-axis represents CNV log(ratio) (upper lane) and allele difference

(lower lane). (**B**) Penetrance plot of CNVs in early (top) and late passage tumors (bottom). x-axis represents chromosomes, y-axis represents proportion of tumors harboring chromosomal gains (green) and losses (red).

A

	A8 CFP	A8 Tomato	H1	H2	H3	H4	H5	H6
Early passages	0/5	0/5	1/5	0/5	1/4	1/5	0/5	1/5
Late passages	0/6	0/6	2/6	4/6	4/5	4/4	0/6	4/6

B

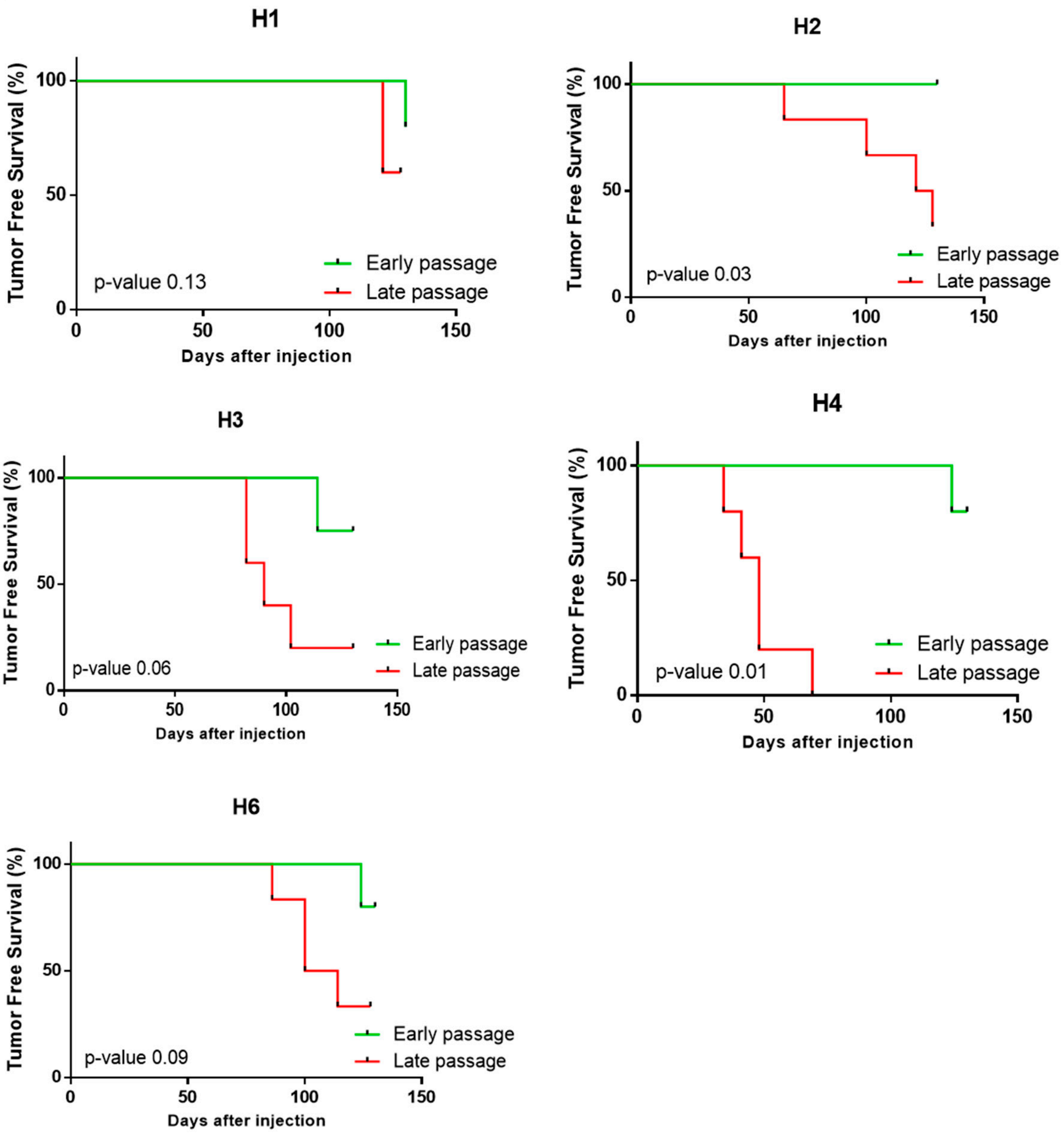

Figure S3. Late passage cell lines developed more tumors than early passage cell lines. (**A**) Mice with tumors developed after 130 days post-injection on total number of mice injected. (**B**) Tumor free survival curve for each hybrid cell lines with tumor development capacity comparing early and late passage cell lines.

A

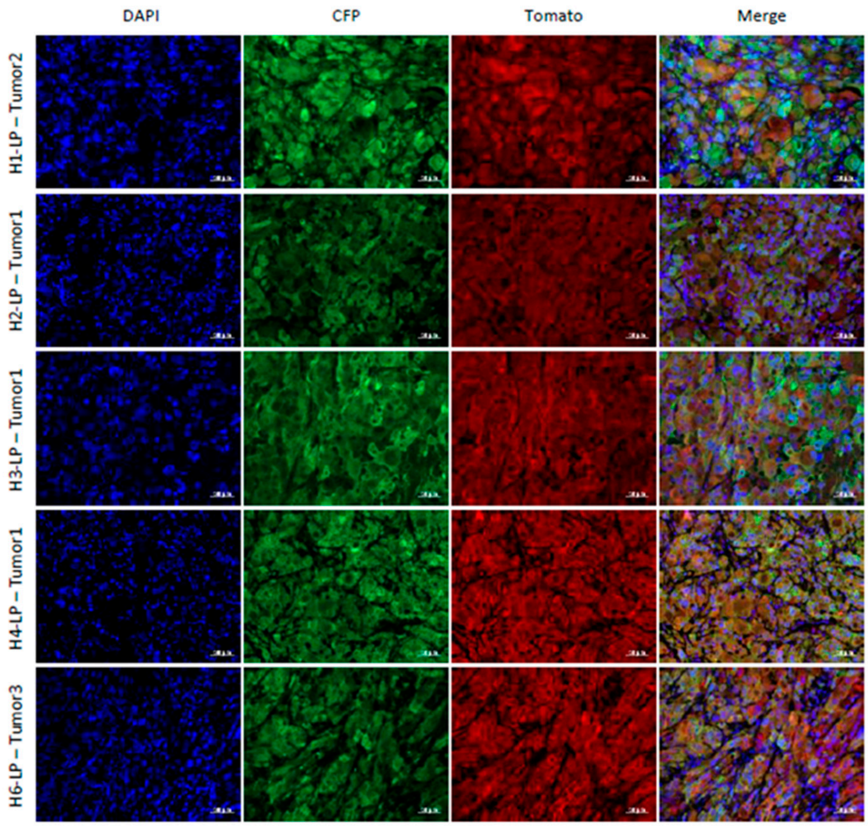
DAPI
CFP
Tomato
Merge
H1-LP – Tumor2
H2-LP – Tumor1
H3-LP – Tumor1
H4-LP – Tumor1
H6-LP – Tumor3

B

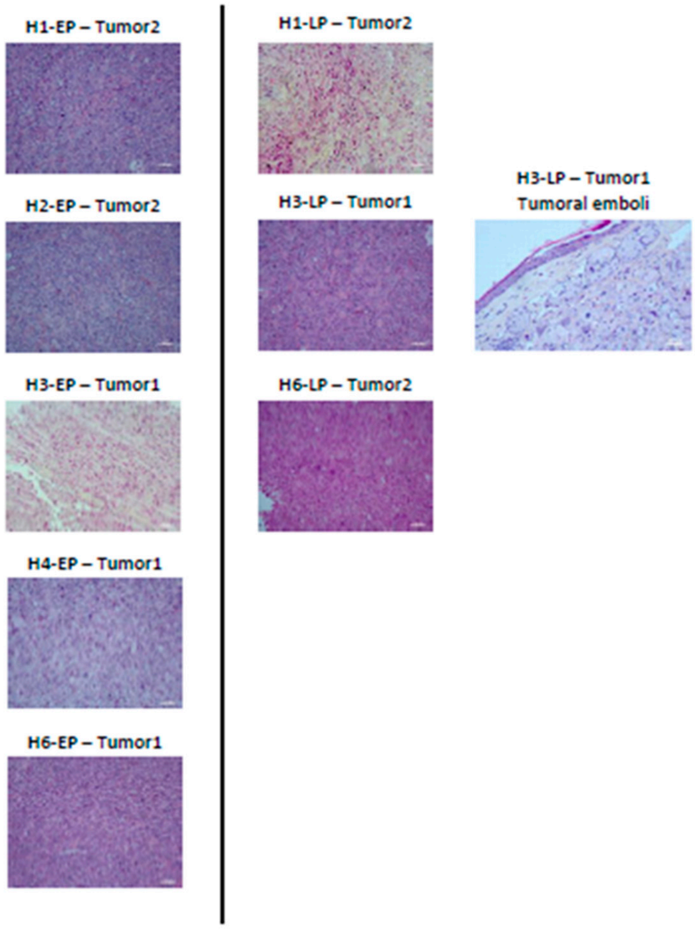
H1-EP – Tumor2
H2-EP – Tumor2
H3-EP – Tumor1
H4-EP – Tumor1
H6-EP – Tumor1
H1-LP – Tumor2
H3-LP – Tumor1
H6-LP – Tumor2
H3-LP – Tumor1
Tumoral emboli

Figure S4. Characterization of hybrid tumors developed in mice. (**A**) Immunofluorescence (CFP and tdTomato) of hybrid tumors. Scale bare = 50 µm. (**B**) Hematoxylin and eosin staining of hybrid tumors. Scale bar = 100 µm.

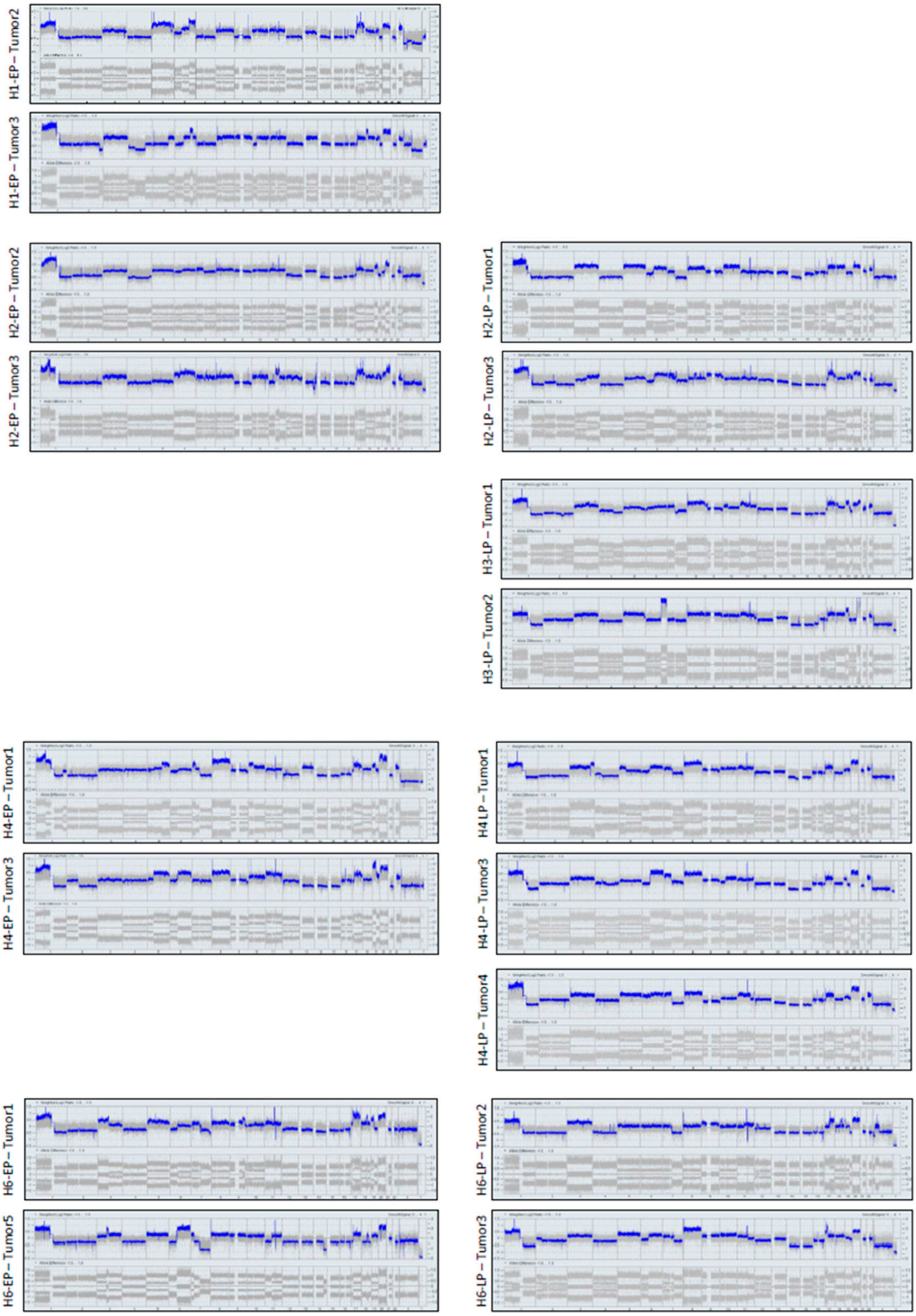

Figure S5. Genomic profiling of EP (left) and LP (right) hybrid tumors. x-axis represents chromosome 1 to Y; y-axis represents CNV log(ratio) (upper lane) and allele difference (lower lane).

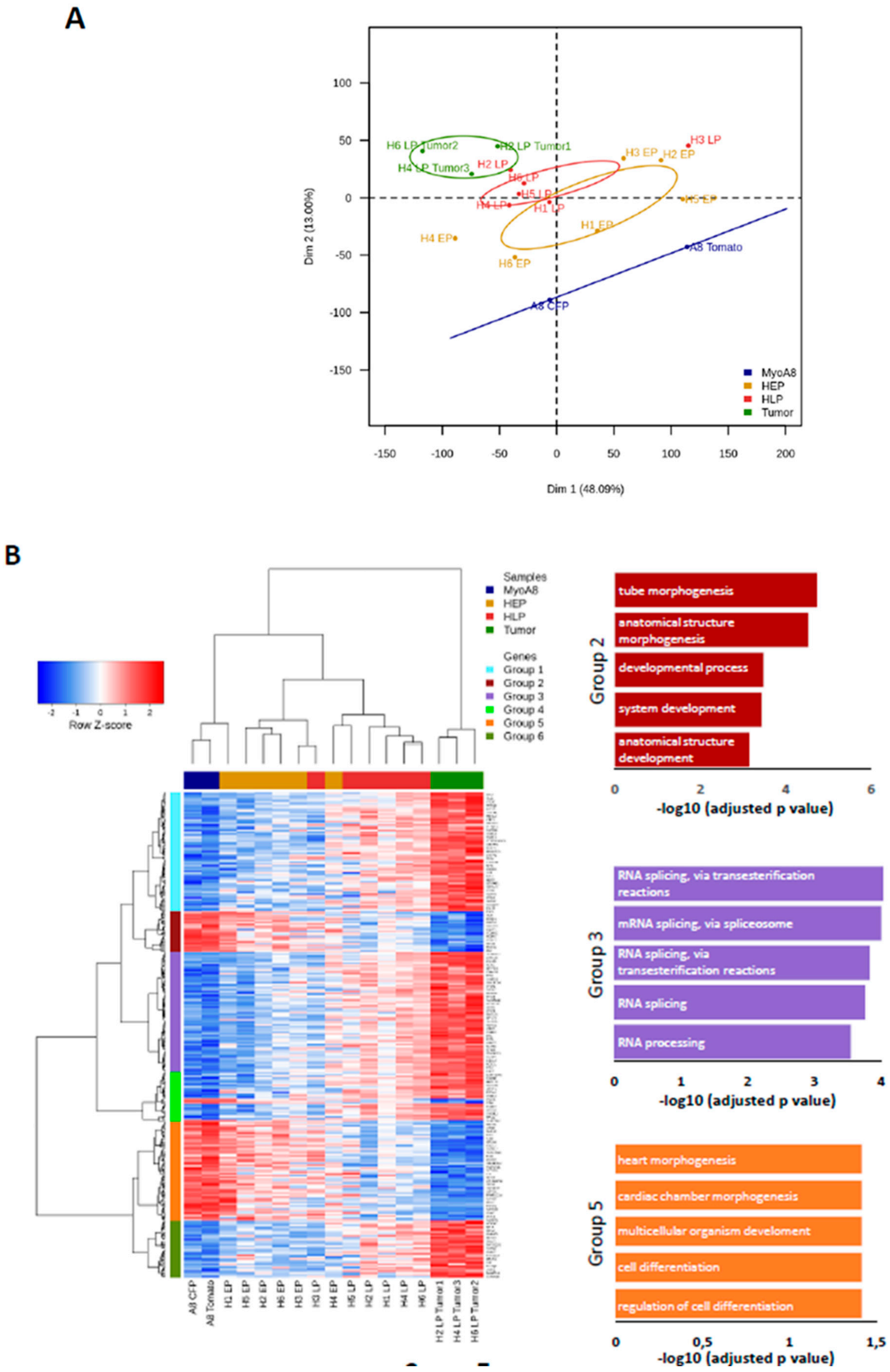

Figure S6. Transcriptomic program is remodeled toward oncogenesis. (A) PCA analysis. x-axis corresponds to dimension 1; y-axis corresponds to dimension 2. (B) Cluster analysis of Z-score expression data (left). Functional enrichment analysis with Gene Ontology (right).

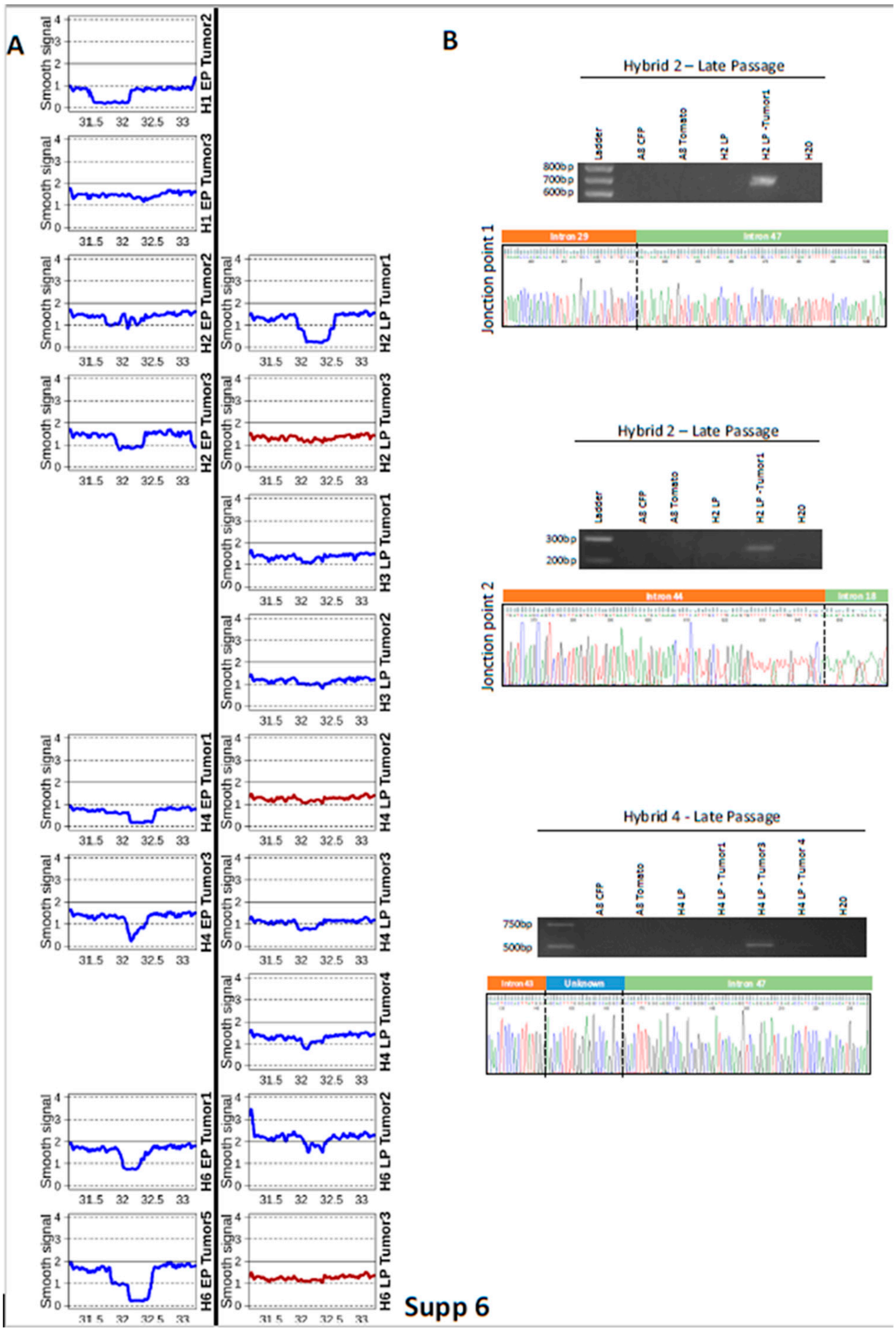

Figure S7. Identification and validation of *DMD* deletions in hybrid tumors. (**A**) Smooth signal (average log2(ratio)) of array CGH for *DMD* gene. Left panel corresponds to EP tumors and right panel to LP tumors. Blue lines are for tumor with detected *DMD* deletion by array-CGH, and red lines for tumor without *DMD* deletion observed. (**B**) Agarose gel (upper panel) and electropherograms obtained by Sanger sequencing (lower panel) showing PCR products of fusion points after break points in *DMD*.

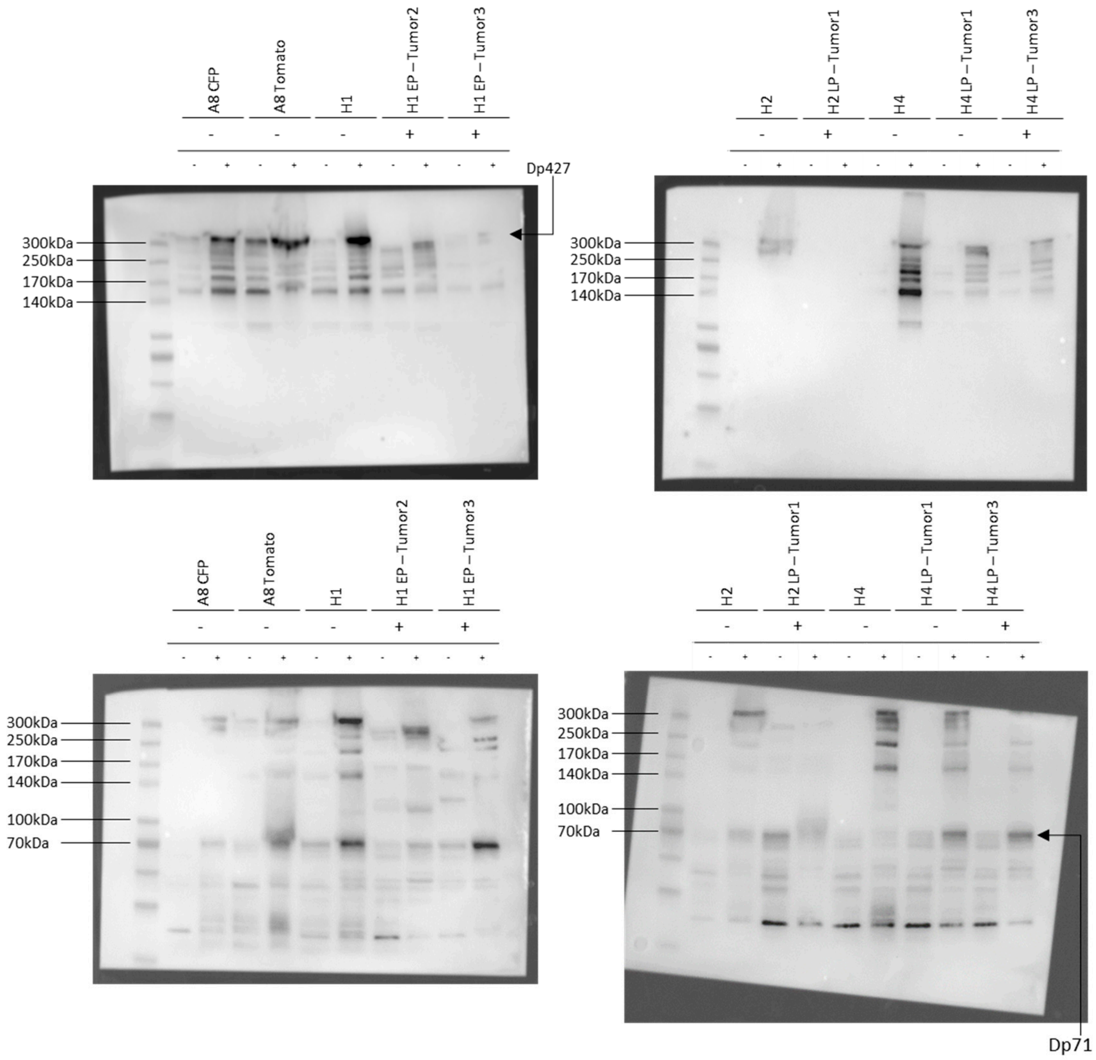

Figure S8. Signal detection on the whole membrane shown on Figure 6A. Dp427 and Dp71 expression in parental and hybrid cell lines. Actin was used as loading control.

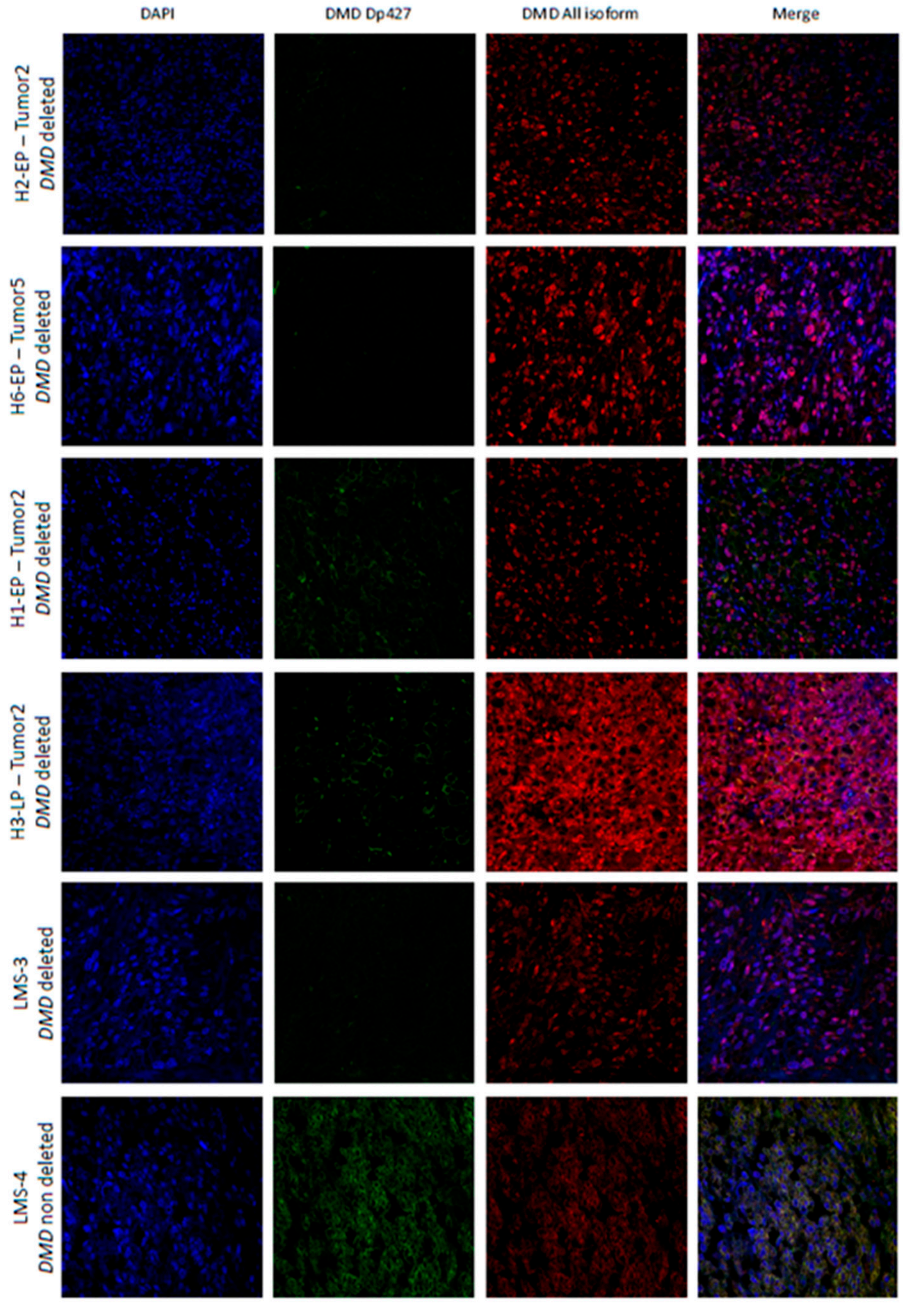

Figure S9. Detection of Dp427 and other dystrophin isoforms by immunofluorescence analysis. Green fluorescence corresponds to Dp427, red to all dystrophin isoform and blue is DAPI to detect nucleus.

Table S1

Group 1		Group 2	Group 3		Group 4	Group 5		Group 6
NFYC	NINJ1	ENC1	MFN2	ZNF259	EIF3I	CSRNP2	EPN2	SPNS2
TTC4	SHMT1	SPRY2	ZCCHC17	RNF220	PSMB7	RBMS2	ZFP90	NFKBIE
NADK	FRG2B	TNS1	LYPLA2	PPIE	PARK7	HERPUD2	SMAD6	RBKS
DFFA	FRG2	PEX11B	LRRC47	MED24	CDC20	ARSB	TMEM169	RELB
MED8	UBIAD1	BACE1	ADPRHL2	TAPBP	ATP6V0B	ATP8B2	MLPH	SREBF1
YRDC	C1orf109	PDGFA	KLHL21	ATAD3A	SH3GL1	ZADH2	AGTR1	GNAZ
NCDN	AP4B1	LAMB1	EFHD2	RPA2	MRPL20	FOXN3	KCNJ15	CPT2
TRIM25	NFS1	PITX2	UQCC	HDAC1	EBNA1BP2	FZD7	PPAPDC1A	NMNAT1
CMTM7	TOE1	CNOT11	SLC25A10	TMEM222	SZRD1	FZD1	STK32B	MED26
KAT2A	HOXB4	TTL	MRPS23	WBP2	C1QBP	NTM	CASQ2	MKNK1
MAP3K14	DHRS7B	FARP1	AATF	SNRNP40	NUDC	DLC1	SGCG	ORC1
AKAP8L	ABR	DDR1	C11orf48	PSMB2	MRPL37	CDH11	MYH7	TRAF1
CLUH	LPCAT3	COL4A2	SMIM4	UBE2J2	NOP56	ADRA2C	TCF7L1	SLC38A3
FMNL1	KRI1	COL4A1	PPIH	BRE	CDC25B	LDOC1	PRRX2	RARRES3
FGGY	AKAP8	FKBP9	DPH2	RER1	DDOST	COL11A1	CDH4	MANEAL
YIPF1	MED9	SLC20A1	TOMM34	PEF1	ATPIF1	TWIST2	SORBS2	ASTN2
NUBP1	AKAP1	PXDC1	GTF2F1	PRPF8	NDUFB4	PTPRM	MATN3	CCDC103
DHDDS	GRIPAP1	CTHRC1	TRAPPC4	WDR77	PRDX4	TNFAIP8L3	CGB2	HMHA1
BRD8	LDLRAP1	RHOB	NABP2	EMC3	STIP1	GNG2	IL15	MORN1
PTPMT1	UBXN11	ANTXR1	SF3A1	NOP16	PSMC5	PKIA	DISC1	KIAA1217
DDX54	SLC2A8	FSCN1	CIRH1A	SDHB	LRRC59	TSHZ1	MEIS1	DDO
SUPT6H	LGMN	IGFBP3	SRSF4	SF3A3	PSAT1	BMPR2	GABBR1	MTUS1
GCAT	PCBD1	FN1	CASC3	MRPS15	COL5A1	PTPN21		OSR2
ABHD8	ASAP3		FN3KRP	TRAPPC3	RTN4	TMEM185B		IL34
NME1-NME2	PLA2G16		C3orf37	TMEM106C	RPS8	SPRED2		CD33
HMGCL	LRIG1		FTSJ3	WASF2	RPL11	CCDC71L		KCNQ4
CTNNBIP1	METTL7B		PGAM5	AK2	ENO1	DGKA		IRAK2
ST6GALNAC4	TRIM47		SLC25A38	EIF4A3	SERPINE1	SFT2D3		ASIC1
NFKB2	PSMB9		DNAJC11	PGM1		CCIN		PPAN-P2RY11
WRAP73	SLC12A7		CCDC137	NOC2L		IL11		BAIAP2L1
TAP2	MT1F		NOP2	MRTO4		ZBTB39		TMCO4
PEX14	ENHO		OGFR	ERI3		IGF1R		TSPAN18
KTI12	SFRP5		BSDC1	RCC1		THUMPD2		
MYBBP1A			STAT3	KIF2C		ARHGAP31		

© 2020 by the authors. Submitted for possible open access publication under the terms and conditions of the Creative Commons Attribution (CC BY) license (http://creativecommons.org/licenses/by/4.0/).
